# Left Ventricular Free Wall Rupture in Broken-Heart Syndrome: A Fatal Complication

**DOI:** 10.7759/cureus.11316

**Published:** 2020-11-03

**Authors:** Amna Al-Tkrit, Andrew Mekaiel, Mohammad Aneeb, Firas Alawawdeh, Aditya Mangla

**Affiliations:** 1 Internal Medicine, Jamaica Hospital Medical Center, Queens, USA; 2 Cardiology, Jamaica Hospital Medical Center, Queens, USA

**Keywords:** takotsubo cardiomyopathy, takotsubo syndrome, broken-heart syndrome, stress-induced cardiomyopathy, cardiac rupture, ventricular free wall rupture, persistent st-segment elevation, complication, acute st-elevation myocardial infarction

## Abstract

Takotsubo cardiomyopathy is usually a transient condition and is treated conservatively. It is rarely associated with ventricular free wall rupture, a fatal complication of the disease described in this report. Cardiothoracic surgery performed emergent ventricular wall repair; however, treatment was unsuccessful, and the patient expired.

## Introduction

Takotsubo cardiomyopathy, also known as broken-heart syndrome, is a form of non-ischemic cardiomyopathy that is typically triggered by emotional or physical stress. It is a reversible condition that responds well to conservative treatment. However, it may sometimes be associated with the development of potentially life-threatening complications. Left ventricular free wall rupture is one such serious complication that occurs extremely rarely. Less than 25 cases of Takotsubo cardiomyopathy-induced left ventricular free wall rupture have so far been reported. In this report, we present a case of left ventricular free wall rupture that developed in a patient with Takotsubo cardiomyopathy and resulted in sudden cardiac arrest and death of the patient

## Case presentation

A 77-year-old woman with a history of hypertension and type II diabetes presented to the emergency department of our hospital for the evaluation of a recent episode of sudden syncope. On presentation, the patient reported severe chest pain that was retrosternal in location and was associated with nausea and vomiting. The patient reported that she was under extreme stress due to the sudden accidental death of a family member.

In the emergency room, the patient was hypotensive, with a blood pressure of 70/40 mmHg. Her heart rate was 110 beats/min, and her respiratory rate was 15 breaths/min. Physical examination was remarkable for cool, clammy extremities, diaphoresis, and faint peripheral pulses. Electrocardiography showed ST-segment elevation in the anteroseptal leads, and cardiac troponin levels were found to be elevated (3.390 ng/mL). A chest X-ray revealed an enlarged cardiac silhouette.

**Figure 1:**
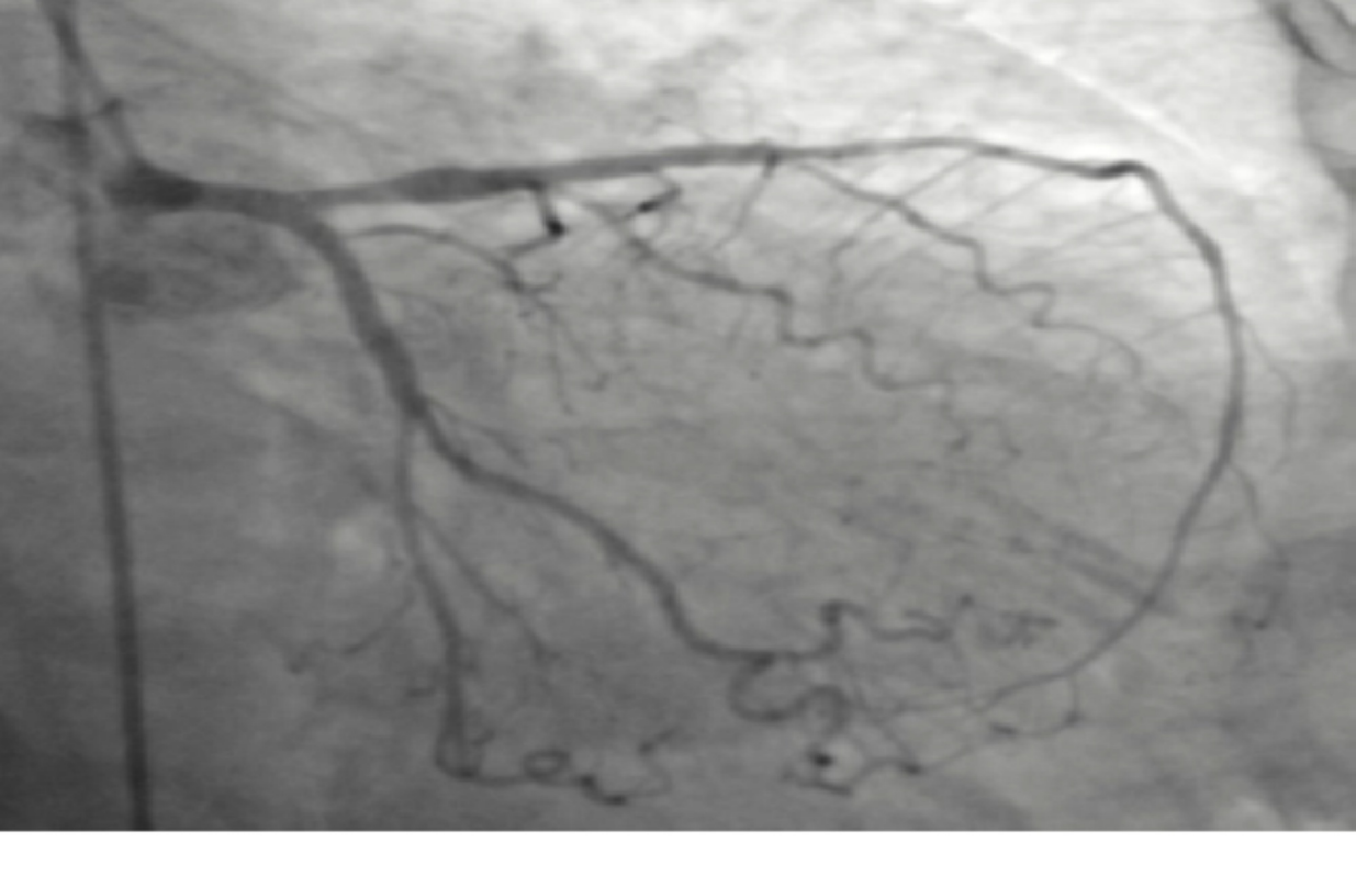
Coronary angiogram showing non-occlusive vessel disease with adequate perfusion.

**Figure 2:**
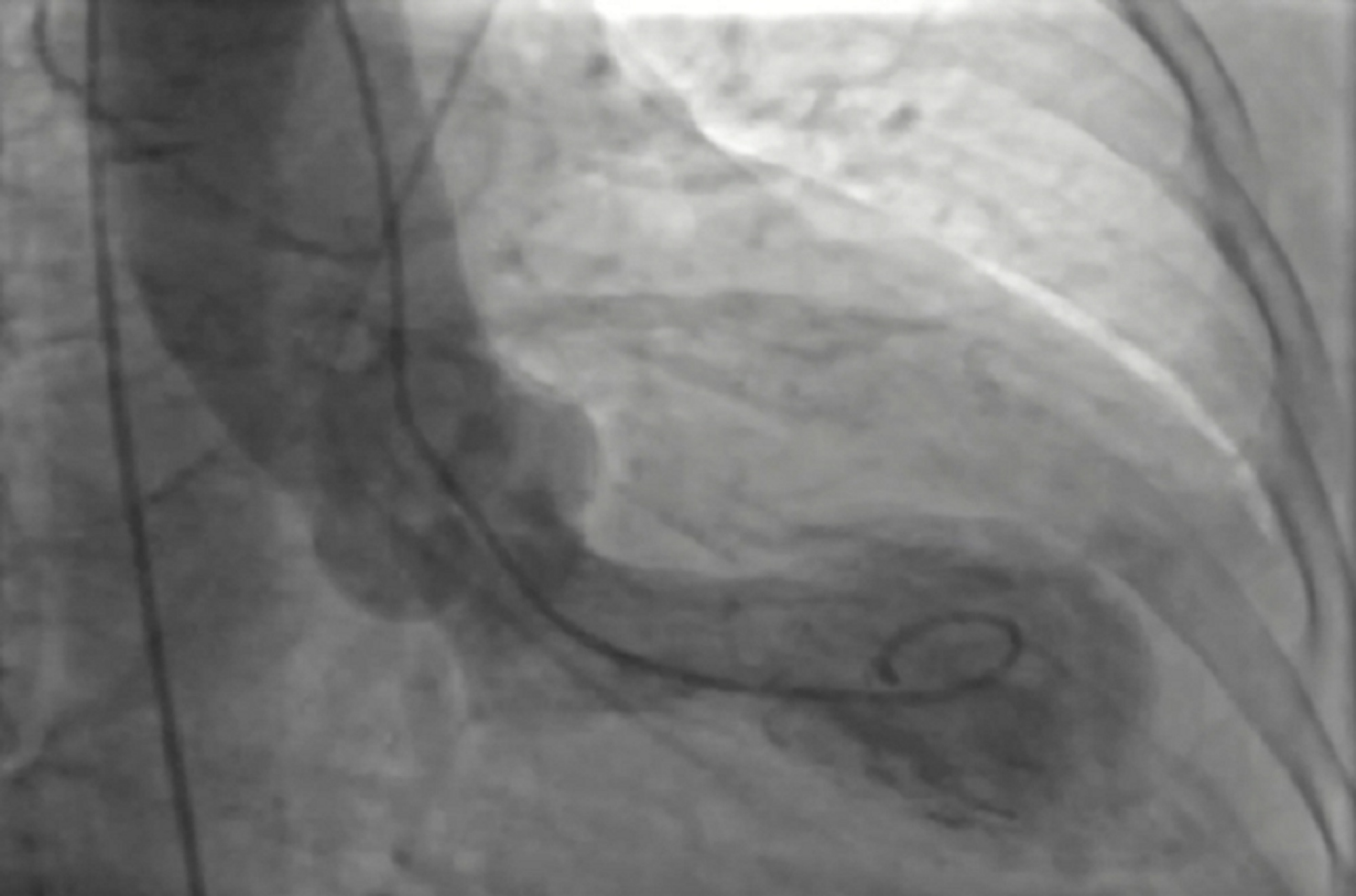
Coronary angiogram showing apical ballooning.

The patient was started on vasopressor therapy to manage cardiogenic shock, and emergency cardiac catheterization was performed. The findings of cardiac catheterization included non-occlusive coronary artery disease, severe anterolateral hypokinesis, apical dyskinesis, and apical ballooning with an estimated left ventricular ejection fraction (LVEF) of 20%. The findings were suggestive of a diagnosis of Takotsubo cardiomyopathy (Figures 1-2).

**Figure 3:**
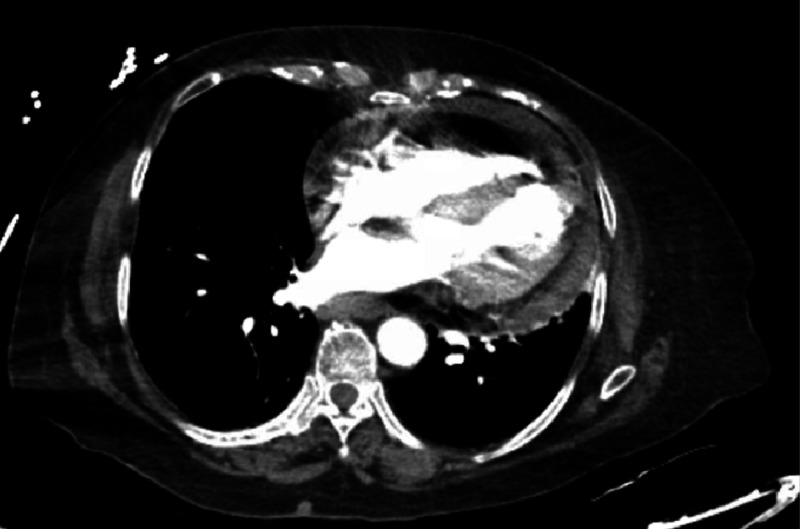
CT chest angiogram showed complex pericardial effusion compatible with hemopericardium.

The patient continued to have severe, persistent chest pain, accompanied by the development of severe back pain. CT angiography of the chest showed complex pericardial effusion, consistent with hemopericardium (Figure 3). Some extravasation of contrast from the left ventricle into the pericardial space was noted, and surgery was consulted based on suspicion of ventricular wall rupture. Emergency cardiothoracic surgery was performed that confirmed the presence of left ventricular free wall rupture, which was treated with large patch repair.

Following the surgery, the patient’s condition deteriorated rapidly, and she went into cardiac arrest. Extensive trials of cardiopulmonary resuscitation were performed but failed to revive the patient, and she eventually expired.

## Discussion

Takotsubo syndrome, also known as Takotsubo cardiomyopathy, stress-induced cardiomyopathy, transient apical ballooning syndrome, and broken-heart syndrome, refers to a form of non-ischemic cardiomyopathy, characterized by transient regional systolic dysfunction of the left ventricle resembling an acute myocardial infarction but without any angiographic evidence of occlusive coronary artery disease or acute plaque rupture [1]. The term “Takotsubo syndrome” was first introduced in a Japanese publication by Dote and Sato in 1990 and 1991. The publication contained a case series of five Japanese patients, the first of which was managed in the Hiroshima City Hospital in 1983. The patient was a middle-aged woman who presented with a clinical and electrocardiographic picture consistent with acute myocardial infarction. However, she had a normal angiographic appearance of the coronary arteries and an unusual appearance of the left systolic ventriculogram with a narrow neck and apical ballooning during systole, which resembled an octopus trap, “takotsubo” in Japanese (tako = octopus, tsubo = a pot). Interestingly, complete resolution of the wall motion abnormalities occurred within two weeks. In the following decade, more women with Takotsubo syndrome, following an acute physical or emotional stress, were reported in Japan. Thus, it was initially assumed that the syndrome only affected people of Asian descent. However, it soon gained international awareness, when the first patient case series in a Caucasian population was published [2,3].

The incidence of Takotsubo cardiomyopathy appears to be increasing worldwide, which is most likely due to an increased awareness of the condition and more widespread use of early invasive coronary angiography; however, it remains an underdiagnosed disease. It has been reported to occur in up to 2% of all patients presenting with the clinical manifestations of acute coronary syndrome (ACS). Data show that approximately 90% of these are women of postmenopausal age. However, the syndrome can occur in both genders and has been described in all age groups, including children and young adults. The prevalence of Takotsubo cardiomyopathy in men appears to be higher in inpatients, suggesting that physical stress, such as a critical medical illness, may play a role in the progression of the disease. According to some reports, the prevalence of this condition is lower in Hispanics and African Americans. Takotsubo cardiomyopathy may recur and the rate of recurrence has been reported to be between 0% and 22% [4,5].

Emotional and physical stress are established triggering factors for the development of broken-heart syndrome. Physical triggers may include exacerbation of medical conditions, such as acute neurological disorders, acute respiratory failure, etc. Examples of emotional triggers are panic, fear, anxiety, grief due to the loss of a loved one, and stress due to interpersonal conflict. However, emotional stress may not always be negative, as positive emotions from joyful or socially desirable events, e.g., a wedding, a surprise birthday party, or a positive job interview, may also lead to the development of Takotsubo cardiomyopathy, a situation that occurs in 1.1% of Takotsubo syndrome and has been described as “happy heart syndrome” [6]. Physical triggers have been found to be more common in men; whereas, emotional triggers are more frequently seen in women. Recent data show that physical triggers are more commonly associated with Takotsubo cardiomyopathy than emotional triggers, and maybe independent predictors of in-hospital complications [7].

The exact pathophysiology of Takotsubo cardiomyopathy is not yet understood; however, sympathetic overstimulation seems to play a central role. An antecedent physical or emotional stressor may result in a sudden surge in the catecholamine levels that can induce left ventricular dysfunction through a variety of proposed mechanisms. For instance, it has been proposed that reversible ventricular dysfunction may be caused by intense multivessel coronary spasm and subsequent regional myocardial stunning, secondary to catecholamine-induced augmentation of adrenergic nerve activity. Another putative mechanism is coronary microvascular dysfunction, as several studies have found abnormal coronary microcirculatory disturbances in patients with Takotsubo cardiomyopathy. The possibility of cardiotoxicity from the catecholamine surge has also been suggested. High circulating levels of catecholamines may induce sarcoplasmic calcium overload, which can lead to impaired cardiac contractility and contraction band necrosis. Activation of myocardial survival pathways is another proposed pathophysiologic mechanism, which suggests that during the catecholamine surge, excessive levels of catecholamines may stimulate myocardial β-adrenoceptors, resulting in a switch from Gs to Gi protein pathway, with a subsequent negative ionotropic effect [8-10]. Recently, the most accepted mechanisms include catecholamine-induced cardiotoxicity and coronary microvascular dysfunction [1].

The clinical presentation of Takotsubo cardiomyopathy usually mimics ACS. Chest pain remains the most common initial presentation and is reported in 50% to 100% of the cases at the onset. Dyspnea is another common presenting symptom and may be seen in 7%-60% of the cases. Sometimes, patients may present directly with heart failure, manifesting as acute pulmonary edema or cardiogenic shock. Palpitations, presyncope or syncope, usually associated with arrhythmia, cardiorespiratory arrest, or sudden cardiac death may also be the initial forms of presentation. In approximately 2%-20% of the patients, Takotsubo cardiomyopathy has been reported to be asymptomatic initially [11].

ST-segment elevation is the most common electrocardiographic abnormality, which is found in 44% of the cases and resembles an ST-elevation myocardial infarction (STEMI). The ST-segment elevation is seen typically in the V2-V5 leads and in leads II and aVR. T-wave inversion may also sometimes occur. Other EKG findings that may be present include QT interval prolongation, Q waves, ST-segment depression, or left bundle branch block. The EKG changes are dynamic and exhibit a temporal evolution. The typical pattern is an initial ST-segment elevation, followed by its resolution, with subsequent appearance of progressive T-wave inversion and QT prolongation. Serum troponin and creatine kinase-MB (CK-MB) levels are usually increased, although the levels tend to be lower than in ACS [2,12].

Apical ballooning of the left ventricle is the typical echocardiographic finding seen in patients with Takotsubo cardiomyopathy, which develops as a result of dyskinesia, hypokinesia, or akinesia of the apical and middle segments of the left ventricle and hypercontractility of the basal segments. The LVEF is decreased initially, with values less than 30% in some cases. Left ventricular outflow tract (LVOT) obstruction, mitral regurgitation with or without systolic anterior motion (SAM), and tricuspid regurgitation may also be seen [11,13]. Coronary angiography with left ventriculography is considered to be the gold standard for the diagnosis of Takotsubo cardiomyopathy [14]. Coronary angiography usually reveals normal coronary arteries and ventriculography exhibits the same changes as seen on echocardiography [11]. Cardiac magnetic resonance (CMR) imaging is another important diagnostic tool that can help visualize the regional wall motion abnormalities and can also characterize myocardial damage, such as edema or fibrosis [15].

Takotsubo cardiomyopathy is a transient and completely reversible entity, and the left ventricular function typically becomes normal within a few weeks. However, it may sometimes be associated with potentially life-threatening complications before recovery, and the in-hospital mortality rate may be as high as 5%. Systolic heart failure may be seen in 12% to 45% of patients. It is the most frequently occurring complication in the acute phase and may lead to cardiogenic shock in some cases. Other common complications include left ventricle outflow tract (LVOT) obstruction, mitral regurgitation, cardiac arrhythmias, and systemic thromboembolism. Ventricular free wall rupture is an infrequent complication associated with Takotsubo cardiomyopathy and has an incidence rate of 0.5% [16,17]. The first case of fatal left ventricular wall rupture in a patient with Takotsubo cardiomyopathy was reported in 2004, and to our knowledge, only about 20 cases have been reported since then [18,19]. Risk factors associated with ventricular free wall rupture include advanced age, female gender, hypertension, and persistent ST-segment elevation. Higher levels of troponin and creatine phosphokinase (CPK), elevated left ventricular intramural pressure, and increased left ventricular wall stress along with high LVOT gradient are some of the other predictors of ventricular free wall rupture in patients with Takotsubo cardiomyopathy. The histopathological findings of myocardial tissue biopsy include contraction band necrosis exhibiting increased eosinophilic staining, mononuclear lymphocytic infiltration, and localized areas of fibrosis at the rupture site, thus indicating that catecholamines play a significant pathogenic role in Takotsubo cardiomyopathy-induced ventricular free wall rupture [17,20].

Currently, there is no standardized protocol for the treatment of patients with Takotsubo cardiomyopathy. All patients should be admitted to an acute cardiac unit with continuous EKG monitoring, imaging, and cardiac catheterization capabilities [20]. The management is generally conservative with a focus on relieving the physical or emotional stress that triggered the condition. However, intensive management may be required in case of acute complications, such as heart failure and cardiogenic shock. The management of heart failure in Takotsubo cardiomyopathy is usually the same as that for heart failure from other causes. An exception to this is LVOT obstruction, in which case, preload and afterload reduction should be avoided. Beta-blockers have been suggested for the prevention of progression and recurrence of Takotsubo cardiomyopathy, and angiotensin-converting enzyme (ACE) inhibitors or angiotensin receptor blockers (ARBs) have been associated with improved survival. However, some studies have failed to show the survival benefits of these medications. Treatment with beta-blockers has, however, been reported to be associated with a lower incidence of cardiac rupture. The treatment of choice for cardiac rupture in patients with Takotsubo cardiomyopathy is urgent surgical repair [19].

The prognosis of Takotsubo cardiomyopathy is usually favorable and conservative management results in an improvement of left ventricular function in the majority of patients. However, devastating complications, such as ventricular free wall rupture, may occur in some cases and are associated with high mortality rates [19]. Our patient presented with signs and symptoms consistent with ACS and had a history of major emotional stress. Coronary catheterization confirmed the diagnosis of Takotsubo cardiomyopathy, which was complicated by left ventricular free wall rupture, and resulted in the death of the patient despite an urgent surgical repair. This case emphasizes the need for close monitoring and early risk stratification of the patients with Takotsubo cardiomyopathy. Initial EKG findings of persistent ST-segment elevation and other risk factors for ventricular free wall rupture may prove to be valuable parameters in predicting a more serious course of disease progression. Further studies are required to delineate the role of beta-blockers, ACE-inhibitors, and ARBs in the prevention of Takotsubo cardiomyopathy-induced ventricular free wall rupture.

## Conclusions

Takotsubo cardiomyopathy is a relatively underdiagnosed condition that may rarely be associated with potentially life-threatening complications, such as ventricular free wall rupture. Medical literature has a paucity of case reports describing Takotsubo cardiomyopathy-induced left ventricular free wall rupture. Hence, the identification of left ventricular free wall rupture in our patient made this a unique case. Continuous EKG monitoring and risk stratification are critical in improving patient survival. Early diagnosis and immediate cardiosurgical intervention may result in a favorable prognosis. Further studies assessing the role of medical management in the prevention of ventricular free wall rupture in patients with Takotsubo cardiomyopathy are needed.
